# Laser Printer Printed Ion Sources for Ambient Ionization Mass Spectrometric Analysis of Volatiles and Semivolatiles

**DOI:** 10.1021/acs.analchem.4c03157

**Published:** 2024-08-13

**Authors:** Chin-Pao Chiu, Yu-Chie Chen

**Affiliations:** † Department of Applied Chemistry, National Yang Ming Chiao Tung University, Hsinchu 300, Taiwan; ‡ International College of Semiconductor Technology, National Yang Ming Chiao Tung University, Hsinchu 300, Taiwan

## Abstract

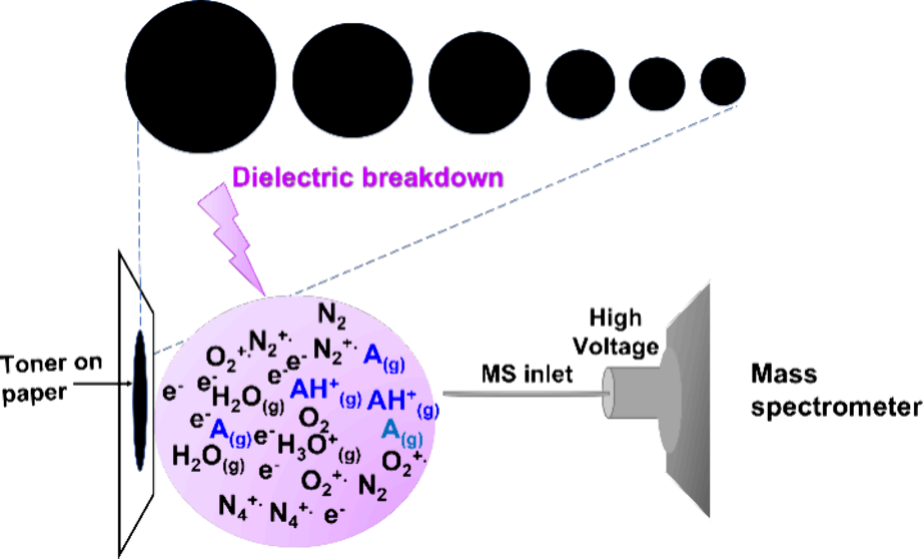

In this study, we demonstrated a facile method to fabricate ion sources using a laser printer for ambient ionization mass spectrometry (MS). Toner spots printed by a printer can readily facilitate ionizing volatile and semivolatile compounds derived from solid or liquid samples for MS analysis. The experimental arrangement involved positioning the toner-printed paper near the inlet of a mass spectrometer, which was subjected to a high electric potential (e.g., −6 kV). Volatile or semivolatile compounds deriving from the sample positioned below the metal inlet of the mass spectrometer were promptly ionized upon activating the mass spectrometer. No direct electrical connection or voltage application was required on the paper substrate. An electric field was established between the toner spot on the paper and the inlet applied with a high voltage to induce the dielectric breakdown of the surrounding air and water molecules. Consequently, ionic species, including electrons and cationic radicals, were generated. Subsequent ion–molecule reactions facilitated the production of protons for ionizing analytes present in the gas phase proximal to the inlet of the mass spectrometer. Deprotonated analytes were detected in the resultant mass spectra when employing the method in negative ion mode. This methodology presents a straightforward approach for analyzing analytes in the gas phase under ambient conditions utilizing an exceptionally uncomplicated experimental setup. In addition, the developed method can be used to detect trace 2,4-dinitrophenol, an explosive, with a limit of detection as low as ∼30 pg.

## Introduction

The first two decades of the 21st century have marked a golden era in the development of ambient ionization mass spectrometry (MS).^1−31^ Plasma-based methods^4−11,27−31^ are one of the main ambient ionization approaches developed in the past two decades. Corona discharge,^32^ glow discharge,^4−6^ dielectric barrier discharge,^9^ and microwave-induced discharge^10^ are commonly employed to generate plasma. However, these plasma-based ionization methods require several accessories, such as a metal needle, an inert gas, a high-voltage power supply, or a microwave generator.

Recently, contactless ionization (or field-induced) methods^11−22^ that require only an ionization substrate, such as a carbon fiber^12,17,18^ have successfully facilitated the ionization of analytes derived from solid and liquid samples. These methods rely on the high voltage provided by the mass spectrometer. Additional electric contact on the ionization substrates mentioned above is not required. These approaches are “contactless,” and a floating ground^34^ occurs on the ionization substrate. The setup for such contactless ionization methods is quite simple. For example, placing a small piece of carbon fiber^17,18^ close to the inlet of the mass spectrometer readily ionizes semivolatiles/volatiles derived from solid or liquid samples placed underneath the inlet of the mass spectrometer. That is, when the high electric field resulting from between the inlet of the mass spectrometer and the ionization substrate reaches a certain threshold, it can prompt the air molecules surrounding the fiber to undergo dielectric breakdown. Consequently, ionic species were generated to initialize subsequent ion–molecule reactions for the ionizing analytes in the gas phase.

Laser printers utilize toner comprised of carbon black as the printing medium.^35^ Carbon black possesses an electrical conductivity. In light of those previous field-induced approaches,^11−22^ we hypothesized that toner spot printed on paper by a printer could also facilitate the dielectric breakdown of air molecules between the toner spot and the inlet of the mass spectrometer that is subjected to a high voltage. Consequently, the resulting ionic species could initiate a cascade of ion–molecule reactions, thereby ionizing the analyte vapors surrounding the metal inlet of the mass spectrometer. This study demonstrated the viability of employing this method to ionize semivolatile and volatile analytes from standards and real samples. Specifically, explosives such as 2,4-dinitrophenol^33^ were chosen as the target analyte to demonstrate the efficacy of the developed technique for rapidly characterizing suspicious and hazardous compounds in real-world scenarios.

## Experimental Section

### Chemicals and Materials

The details of the chemicals and materials used in this study are listed in the Supporting Information (SI).

### Instrumentation

An HP Laser 150a printer (California, USA) was used to print ion sources. An amaZon SL mass spectrometer (Bruker Daltonics, Germany) was used to acquire all of the mass spectra shown in this work. The ion charge control was set to 100 000 ions, with a maximum acquisition time of 200 ms. The nebulizer was switched off during the MS analysis, and the ion transfer capillary temperature was set to 200 °C.

### Setup of the Developed Ionization Method

**Scheme 1:**
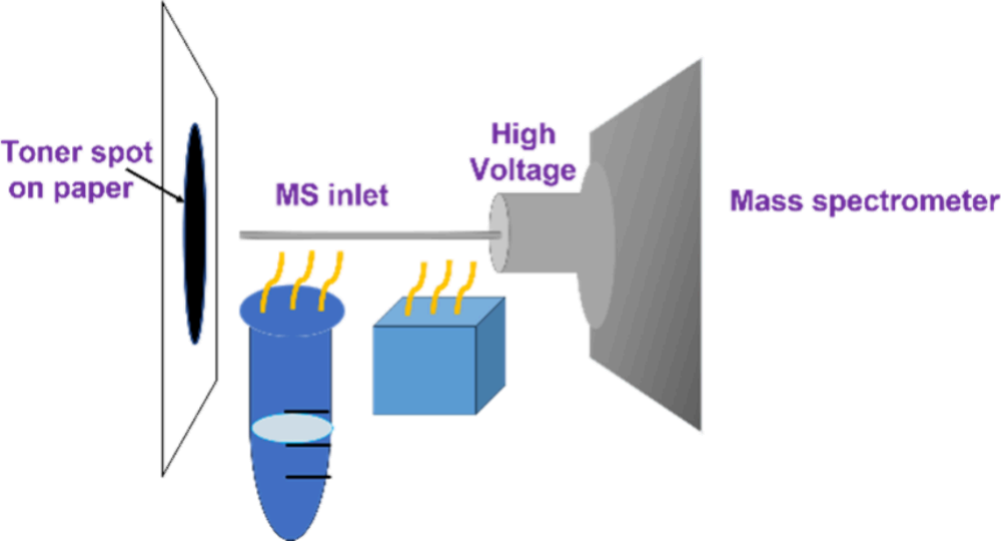
Cartoon Illustration of the Setup of the Developed Ionization Method

Scheme 1 shows a cartoon illustration of our setup. The metal inlet tube was directly attached to the ion transfer capillary with a loose slide-on connection. The tip of the metal tube was rounded and polished to prevent electrical sparking. A piece of paper printed with a toner spot, which was repeatedly printed on the same position five times, was placed close to the inlet of the mass spectrometer. A sample solution was placed underneath the inlet at ∼0.5 cm. The distance between the metal inlet and the paper was ∼1 mm. Mass spectra were immediately acquired when the mass spectrometer was switched on. A metal inlet (length: 3.5 cm; inner diameter: ∼0.5 mm; outer diameter: ∼1 mm) was adapted to the orifice of the mass spectrometer. −6000 V and +6000 V were applied on the orifice when operating in the positive and negative ion modes, respectively.

## Results and Discussion

### Laser Printer Printed Ion Sources

**Figure 1:**
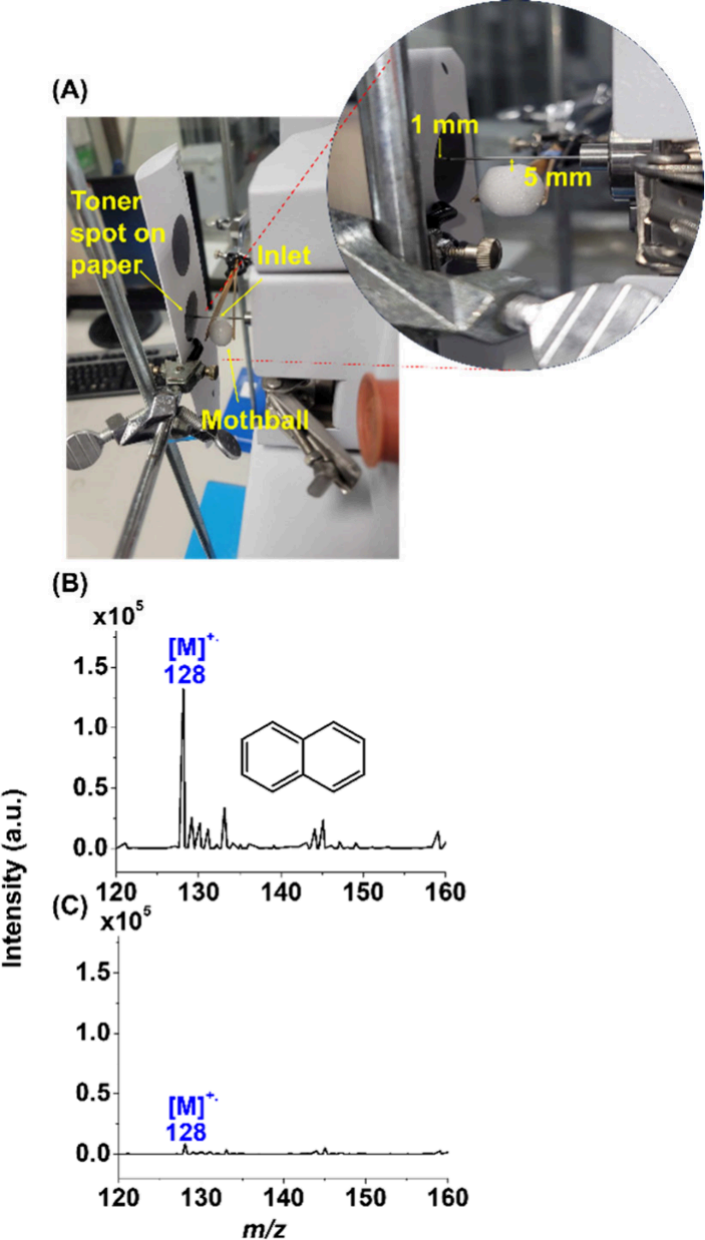
(A) Photograph of the setup of our ionization method. The resulting mass spectra of a mothball by placing it underneath the inlet using our setup obtained (B) with and (C) without a toner-composed spot on the paper that was placed close to the inlet of the mass spectrometer.

Figure 1A shows a photograph of our ionization setup. A piece of paper printed with a toner spot was placed close to the mass spectrometer. A mothball, mainly composed of semivolatile naphthalene, was placed underneath (∼0.5 cm) the metal inlet. Figure 1B shows the resultant mass spectrum. The peak at *m*/*z* 128 derived from the cationic radical of naphthalene was immediately observed in the mass spectrum with the mass spectrometer. Figure 1C shows the mass spectrum of the same sample used to obtain Figure 1B by placing a blank paper close to the inlet of the mass spectrometer instead. Apparently, the peak at *m*/*z* 128 derived from naphthalene was relatively low. The toner, i.e., carbon powder, on the paper can effectively facilitate the ionization of semivolatile analytes derived from the solid sample using our method. It is worth mentioning that the toner spot was obtained by repeatedly printing on the same position five times, as the analyte peak was enhanced with the number of printings (SI Figure S1). However, the paper overheated after more than five printings. Therefore, we used the toner spot created by five printings to facilitate the ionization of analytes using our approach. Nevertheless, the toner spot with a high density of carbon powder could be generated by crossing black lines at the same position on the paper. The intersection point obtained by crossing five black lines worked similarly to those obtained from five-time printing (SI Figure S2). The density of the carbon powder on the toner spot increased with repeated printing. This higher density likely helped generate a stronger electric field between the toner spot and the metal inlet of the mass spectrometer, enhancing the dielectric breakdown process and improving the ionization efficiency for analyte vapor derived from the sample in the condensed phase.

### Optimization of the Experimental Parameters

We further examined the optimal experimental parameters, including the voltage applied on the orifice of the mass spectrometer, the distance between the inlet and the toner-based ion source, and the distance between the inlet and the sample. A mothball was still used as the model sample, and the setup was similar to that shown in Figure 1A. SI Figure S3A–C show the plots obtained by plotting the ion intensity of the peak at *m*/*z* 128 derived from naphthalene versus those three parameters, as stated above. The results showed that the voltage at −6000 V, the distance at 1 mm between the paper and the inlet, and the distance at 0.5 cm between the sample and the inlet could obtain the highest intensity of the peak at *m*/*z* 128 derived from naphthalene. These results indicated that a higher voltage and a smaller gap between the toner spot and the metal inlet could help to provide a sufficiently high electric field. As a result, the dielectric breakdown of the air molecules between the metal inlet and the toner spot for the generation of ionic species could be easily induced. The optimal experimental parameters were used to obtain the results in the following studies.

### Analysis of Semivolatiles and Volatiles

Semivolatiles including ametryn, atrazine, azulene, 2,2-bipyridine, cinnamaldehyde, and 2,4-dichlorophenol were selected as the model analytes using the developed approach. Their corresponding vapor pressure values have been listed in SI Table S1. SI Figure S4 shows the resulting mass spectra obtained by putting the sample solution (2 mL) underneath the metal inlet. Protonated analytes ([M + H]^+^) dominated the mass spectra at the positive ion mode (Figure S4A–E), except that the deprotonated molecule ([M – H]^−^) derived from 2,4-dichlorophenol obtained at the negative ion mode (Figure S4F). The lowest detectable concentration of using our method varied for different analytes (1 μM to 10 nM; SI Table S1). Our method is suitable for detecting trace analytes such as phenols, e.g., 2,4-dinitrophenol, which is an explosive. Thus, we applied trace 2,4-nitrophenol (∼37 pg (20 μL, 10^–8^ M)) to the toner spot on the paper. After solvent evaporation, the paper was positioned near the inlet of the mass spectrometer for MS analysis (inset photograph in Figure S5). The peak at *m*/*z* 183 derived from the deprotonated 2,4-dinitrophenol with its signal-to-noise ratio (S/N) of ∼5 dominated the mass spectrum (Figure S5). The LOD was estimated to be ∼30 pg by considering an S/N of 3, which was comparable to that obtained by using plasma-based ambient ionization MS for the detection of explosives.^33^ However, the setup of our current method was simpler. These results underscore the feasibility of utilizing our method for rapidly characterizing trace semivolatiles, including hazardous compounds.

In addition, volatile organic solvents, including acetone, toluene, and hexanol, could be readily analyzed by using our method. SI Figure S6 shows the resulting mass spectra when these solvents were placed underneath the inlet of the mass spectrometer for MS analysis. Protonated acetone and toluene at *m*/*z* 59 (SI Figure S6A) and 93 (SI Figure S6B), respectively, were observed in the resulting mass spectra. The fragment at *m*/*z* 85 derived from hexanol with a loss of a hydroxyl group appeared in the resulting mass spectrum (SI Figure S6C). Some background peaks at *m*/*z* 143, 157, 170, and 185 derived from the toner resin could be observed in the mass spectrum (SI Figure S7) when no sample was placed underneath the metal inlet of the mass spectrometer. Nevertheless, these background ions were easily suppressed when a sample containing semivolatiles or volatiles was placed underneath the metal inlet. These results indicated that our method could be used to detect volatiles and semivolatiles.

### Putative Ionization Mechanism

Presumably, the ionization processes in the current approach were similar to those that occur in the corona discharge^32^ and plasma-based ionization, such as direct analysis in real time.^5^ We hypothesized that nitrogen and water molecules in the air were ionized under the electric field between the inlet of the mass spectrometer and the toner spot on the paper through dielectric breakdown processes, followed by a series of ion–molecule reactions (see the reactions below). Analyte molecules (A) in the gas phase were ionized through protonation.
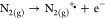

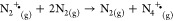

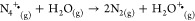

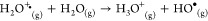

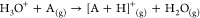


That is, protons were derived from moisture in the air. To validate the putative ionization mechanism, we placed boiling D_2_O underneath the inlet of the mass spectrometer when conducting the MS analysis of the sample solution containing ametryn using the current method. SI Figure S8A and B show the resulting mass spectra of the sample solution containing ametryn obtained before and after, respectively, placing boiling D_2_O underneath the inlet of the mass spectrometer during the MS analysis. The peak at *m*/*z* 228, corresponding to the protonated ametryn, dominated the mass spectrum without D_2_O vapor. After placing the boiling heavy water, the peak at *m*/*z* 229 became the base peak, and some hydrogen–deuterium exchanges also occurred, leading to the peak shifting to higher than *m*/*z* 229. These results validated the proposed ionization processes.

### Analysis of Real Samples

**Figure 2:**
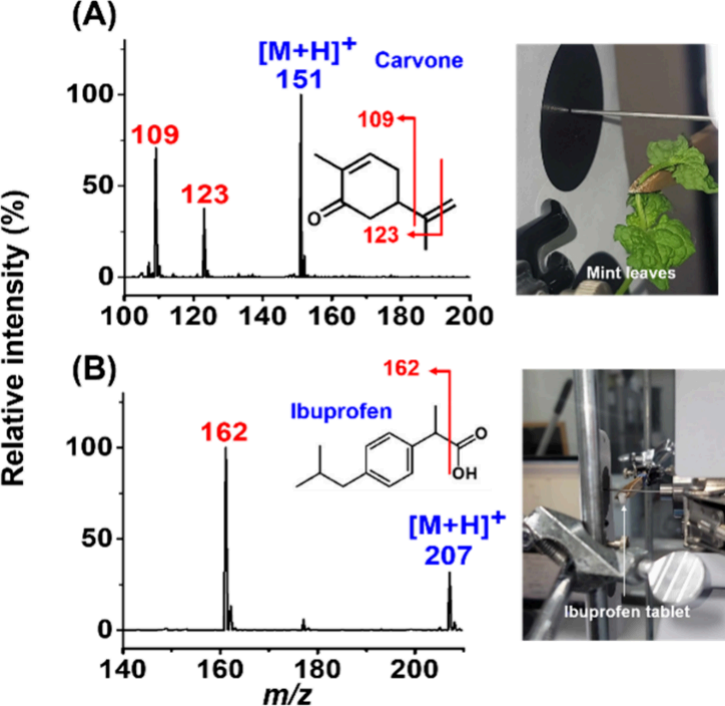
Mass spectra of the samples including (A) mint leaves and (B) a tablet containing ibuprofen obtained by placing the samples underneath the inlet as shown in the photographs on the right-hand side of each mass spectrum.

We further examined whether our method can be used to detect volatiles and semivolatiles from real-world samples. Mint leaves and a drug tablet containing carvone and ibuprofen were positioned beneath the metal inlet for MS analysis using our approach. Figure 2A and B show the resulting mass spectra with accompanying photographs of each setup displayed on the right-hand side. The peaks at *m*/*z* 151 (Figure 2A) and *m*/*z* 207 (Figure 2B) corresponding to protonated carvone from the mint leaves and protonated ibuprofen from the drug tablet, respectively, were observed in their respective mass spectra. Moreover, the fragments (marked in red) derived from carvone and ibuprofen were also observed in the corresponding mass spectra, with the inset structures providing detailed information about these fragments. These results indicated that our method can be effectively utilized to detect primary aroma molecules or semivolatiles derived from real-world samples without requiring any sample pretreatment. Furthermore, the generated fragments aid in identifying the semivolatiles observed in the resulting mass spectra.

### Comparison of the Current Method with the Existing Field-Induced Ionization Methods

We previously have developed several field-induced ionization methods for MS analysis of volatiles and semivolatiles.^15,18,23^ These methods have the features of simplicity and ease of operation. Nevertheless, using a printer to print a toner spot on a piece of paper is relatively easy compared with the previous methods. SI Table S2 lists the comparison of the lowest detectable concentration of semivolatile standards. The lowest detectable concentration of the current approach is comparable to that obtained using an insulating fiber-based ionization approach.^15^ Moreover, the current approach has a lower detectable concentration than those obtained from CFI-MS^18^ and copper wire-coiled metal inlet-based ionization-MS.^23^ Although the standards used to examine the lowest detectable concentration were not the same across these methods,^15,18,23^ those standards that could achieve the lowest detectable concentration were selected at the time.

## Conclusions

We have demonstrated that a laser printer can be used to print paper-based ion sources for ambient ionization MS analysis of volatiles and semivolatiles from either liquid or solid samples. The fabrication of the ion source is simple, whereas the developed method is straightforward and is easily operable. Samples can be analyzed either by being placed underneath the inlet of the mass spectrometer or directly deposited on the toner spot of the paper. The electric field induced between the toner spot printed on a piece of paper and the inlet of the mass spectrometer, applied with high voltage, is enough to cause the generation of ionic species in between through dielectric breakdown. Given the features of simplicity and speed, the developed method is suitable for the high-throughput analysis of samples containing volatiles/semivolatiles. This developed method should be ideal for pairing with a portable mass spectrometer that favors a compact and simple ion source. Furthermore, efforts should be dedicated to further exploring the feasibility of using the current setup to analyze nonvolatile organics.

## Supporting Information Available

Additional experimental details; list of semivolatiles used in this study; comparison of the lowest detectable concentrations; examination of the effects of printing times of toner spots; examination of the intersection points; optimization of the experimental parameters; analysis of semivolatiles; examination of the LOD on the toner spot; analysis of volatiles; blank mass spectrum of the toner spot; examination of ionization mechanisms (PDF)
