# Supplementary material for: Laser Printer Printed Ion Sources for Ambient Ionization Mass Spectrometric Analysis of Volatiles and Semivolatiles
Source: Anal Chem. 2024 Aug 13;96(34):13751–5. doi: 10.1021/acs.analchem.4c03157 (PMC11359382; doi:10.1021/acs.analchem.4c03157)
Supplement: Supplementary file 1 — ac4c03157_si_001.pdf [file ac4c03157_si_001.pdf]

Supporting Information

Laser Printer Printed Ion Sources for Ambient Ionization Mass Spectrometric Analysis of Volatiles and Semivolatiles

Chin-Pao Chiu[1] and Yu-Chie Chen*[1,2]

[1]Department of Applied Chemistry, National Yang Ming Chiao Tung University, Hsinchu 300, Taiwan

[2]International College of Semiconductor Technology, National Yang Ming Chiao Tung University, Hsinchu 300, Taiwan

*Corresponding author

yuchie@nycu.edu.tw

Table of Contents

Additional Experimental Details……………………………………………………S2

Table S1. List of semivolatiles used in this study………………………………S3

Table S2. Comparison of the lowest detectable concentrations ………………………S4

Figure S1. Examination of the effects of printing times of toner spots……………...…S5

Figure S2. Examination of the intersection points……………………………………S5

Figure S3. Optimization of the experimental parameters ……..……………………S6

Figure S4. Analysis of semivolatiles…………………………..…………………S6

Figure S5. Examination of the LOD on the toner spot…….………………………S7

Figure S6. Analysis of volatiles. ……………………………..……………………S7

Figure S7.Blank mass spectrum of the toner spot……………………………………S8

Figure S8. Examination of ionization mechanisms………..………………………S8

References…………………………………………………………………………..S9

Additional Experimental Details

Chemicals and Materials

Azulene and cinnamaldehyde were purchased from Sigma-Aldrich (St. Louis, Mo, USA). 2,2'-Bipyridine was purchased from Alfa Assar (Ward Hill, MA, USA). Ametryn and 2,4-dinitrophenol were acquired from Riedel-de Haën (Seelze, Germany). 2,4-Dichlorophenol was purchased from Acros Organics (Geel, USA). Acetonitrile, methanol, ethanol, toluene, and hexanol were obtained from Merck (Darmstadt, Germany), Macron Fine Chemicals (Center Valley, PA, USA, Echo Chemical (Miaoli, Taiwan), Avantor (Corporate Parkway, PA, USA), and J. T. Baker (Phillipsburg, New Jersey, USA), respectively. Cartridges containing toner were purchased from HP (California, USA). Mothballs (99% naphthalene) and A4 paper were purchased from local shops.

Table S1. List of semivolatiles used in the study.

Analyte	Monoisotopic mass	Structure	Vapor pressure (mmHg)[a] at 25°C	Lowest detectable concentration
Ametryn	227.12	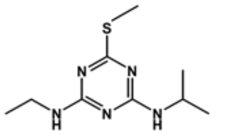	2.74×10^{-6}	10 nM (S/N= 552)
2,2-Bipyridine	156.07	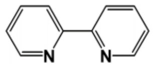	1.30×10^{-5}	10 μM (S/N= 7172)
Cinnamaldehyde	132.06	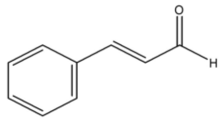	2.89×10^{-2}	1 μM (S/N= 3860)
2,4-Dichlorophenol	161.96	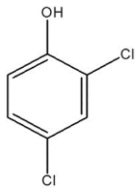	9.00×10^{-2}	0.1 μM (S/N= 180)
2,4-Dinitrophenol	184.01	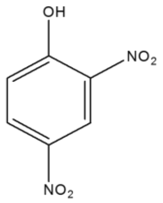	1.20×10^{-5}	10 nM (S/N= 200)
Prometryn	241.14	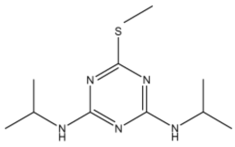	1.24×10^{-6}	10 nM (S/N= 1473)

[a]Vapor pressure values were obtained from https://pubchem.ncbi.nlm.nih.gov/.

Table S2. List of the lowest detectable concentrations of the analytes obtained from the existing field-induced ionization MS.[1-3]

**Ionization method**	**Analyte**	**Lowest detectable concentration***
Carbon fiber ionization	Methyl cinnamate	~1.7 μM[1]
Insulating fiber-based ionization	Ametryn	10 nM[2]
Copper wire coiled metal inlet-based ionization	2.4-Dinitrophenol	10 μM[3]
This work	2,4-Dinitrophenol/ametryn	10 nM

*The sample (2 mL) was placed underneath the metal inlet for MS analysis.

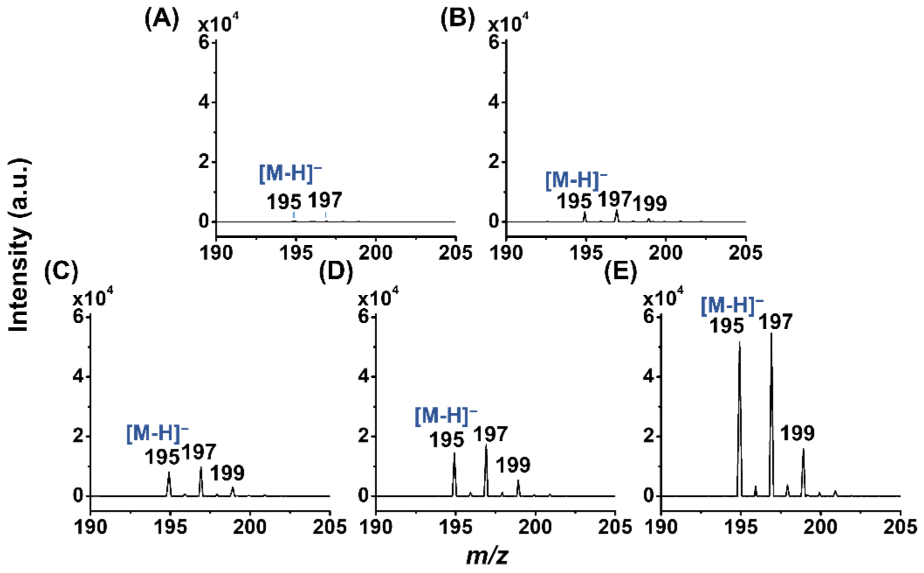

Figure S1. Examination of the effects of printing times on toner spots on paper. Mass spectra of the sample solution (2 mL) containing 2,4,6-trichlorophenol (0.1 mM) obtained by using the toner spots generated by repeatedly printing for (A) one, (B) two, (C) three, (D) four, and (E) five times on paper to assist the ionization of the target analyte using our approach.

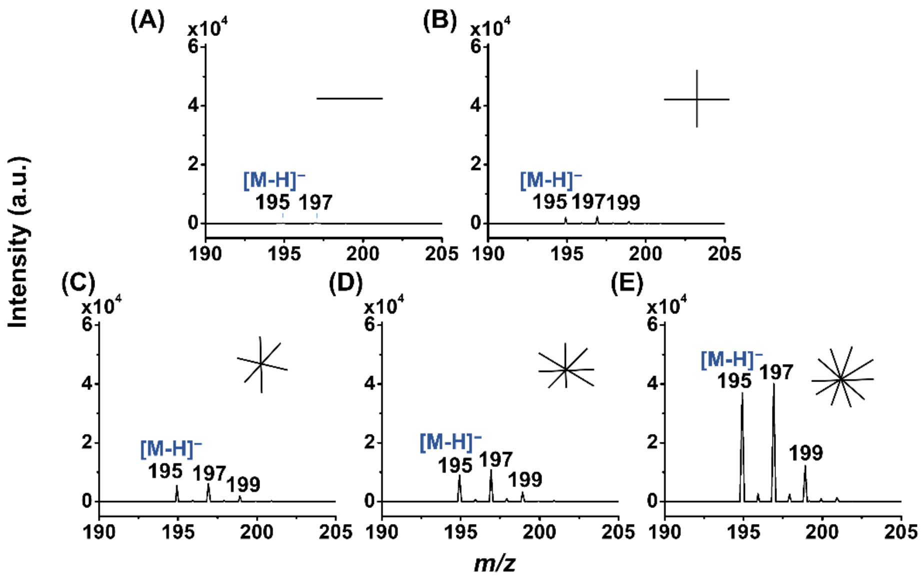

Figure S2. Examination of the intersection points of various numbers of black lines. Mass spectra of the sample solution (2 mL) containing 2,4,6-trichlorophenol (0.1 mM) obtained by using the intersection points of various numbers of black lines obtained by (A) one, and crossing (B) two, (C) three, (D) four, and (E) five black lines (line thickness: 0.2 cm) on paper to assist the ionization of the target analyte using our approach. The insets show the illustrations of how the intersection points were created from different numbers of black lines on paper. The intersection points were placed close to the inlet of the mass spectrometer to assist MS analysis.

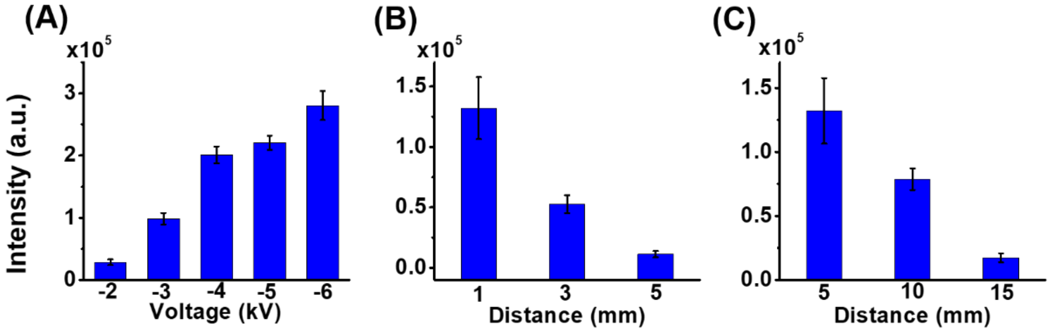

Figure S3. Optimization of the experimental parameters. Examination of (A) the voltage applied on the orifice of the mass spectrometer, (B) the distance between the toner spot on paper and the inlet of the mass spectrometer, and (C) the distance between the surface of the sample and the inlet of the mass spectrometer. A mothball (99% naphthalene) was placed underneath the inlet of the mass spectrometer for MS analysis at the positive ion mode using our method.

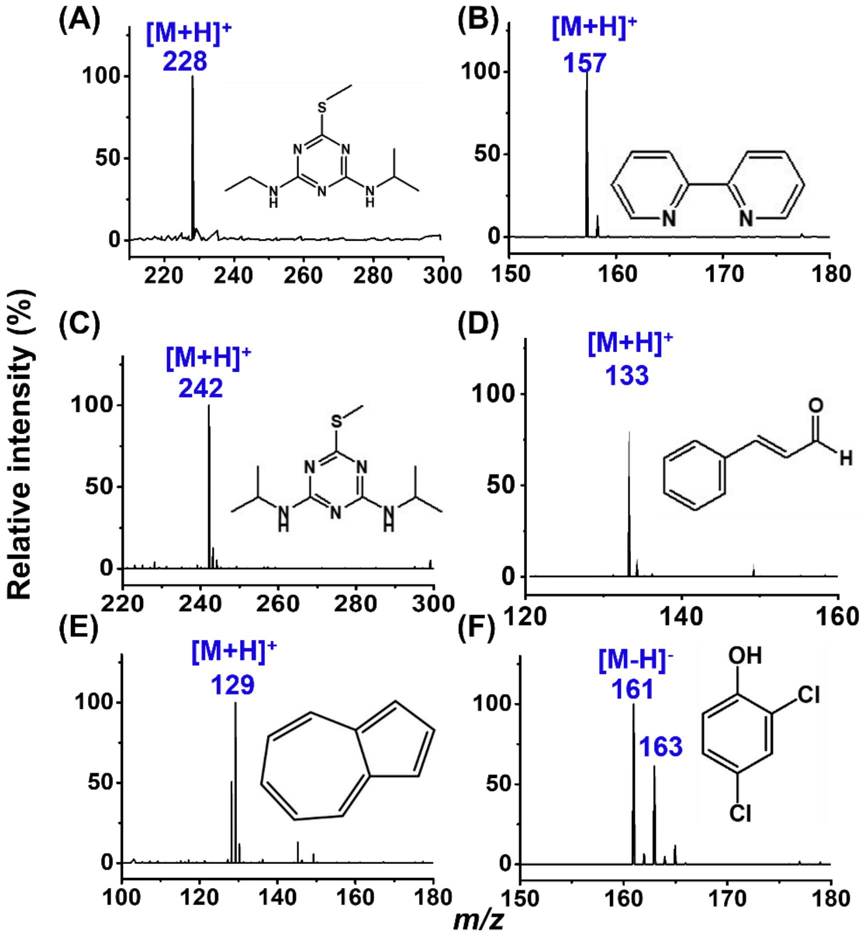

Figure S4. Analysis of semivolatiles. Mass spectra of the sample solutions containing (A) ametryn, (B) 2,2-bipyridine, (C) prometryn, (D) cinnamaldehyde, (E) azulene, and (F) 2,4-dichlorophenol. The concentrations of all the analytes were 0.1 mM.

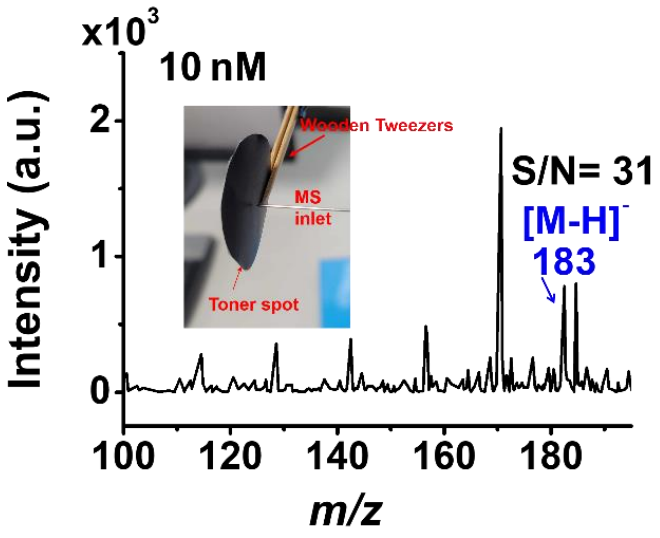

Figure S5. Examination of the LOD on the toner-composed spot. Mass spectrum obtained by depositing a sample solution (20 µL) containing 2,4-dinitrophenol (10 nM) on the toner-composed spot, followed by drying and placing it close to the inlet of the mass spectrometer for MS analysis. The inset photograph shows the setup of the approach. The distance between the toner spot and the inlet of the mass spectrometer was ~1 mm.

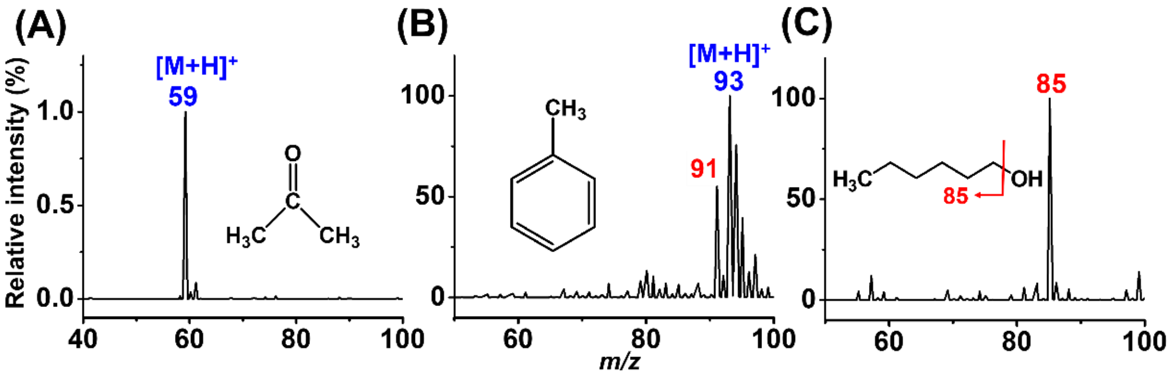

Figure S6. Analysis of volatiles. Mass spectra of the samples containing volatile organic solvents, including (A) acetone, (B) toluene, and (C) hexanol, obtained using our method.

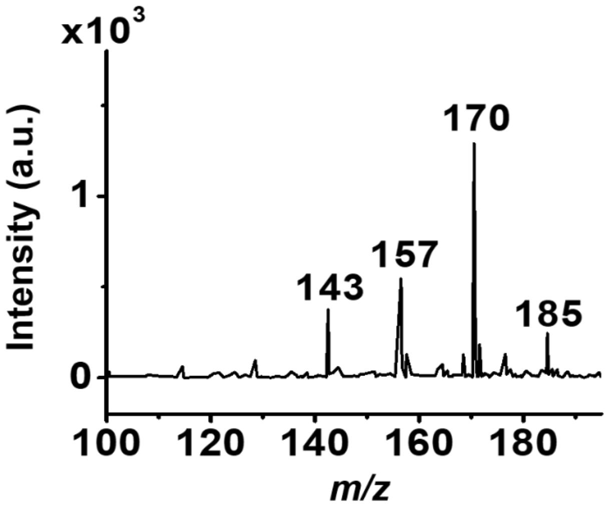

Figure S7. Blank mass spectrum of the toner spot on a piece of paper obtained using our method.

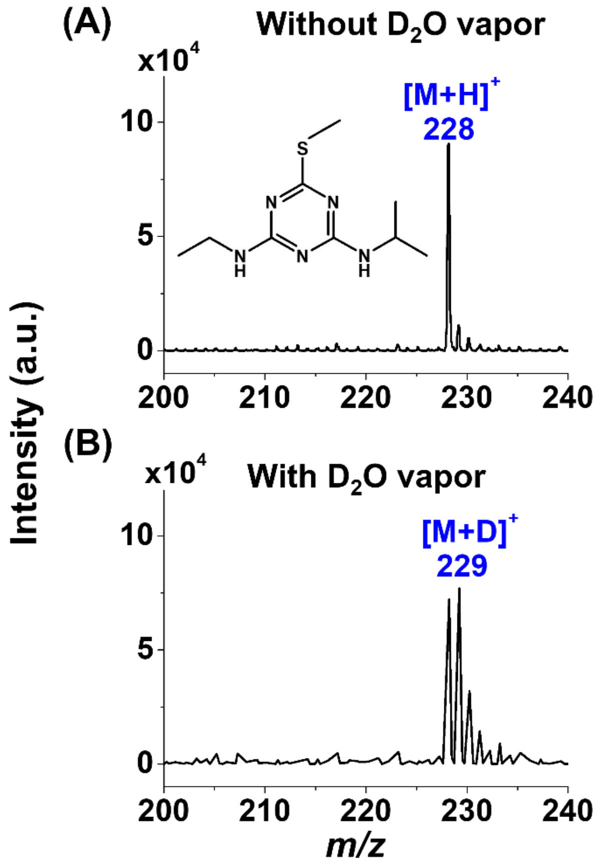

Figure S8. Examination of the ionization mechanism. Mass spectra of the sample (2 mL) containing ametryn (0.1 mM) obtained (A) without and (B) with boiling heavy water undeath the metal inlet of the mass spectrometer using our ionization approach.

References

1. Wu, M.-L; .Chen, T,-Y; Chen, Y.-C., Chen, Y.-C. Carbon fiber ionization mass spectrometry for the analysis of analytes in vapor, liquid, and solid phases. *Anal. Chem.* 2017, 89, 13458–13465

2. Selvaprakash, K. and Chen, Y.-C. Using an insulating fiber as the sampling probe and ionization substrate for ambient ionization–mass spectrometric analysis of volatile, semi-volatile, and polar analytes. *Anal. Bioanal. Chem.* 2022, 414, 4633–4643.

3. Tu, C.-F.; Chen, Y.-C. Utilizing a metal inlet coiled with copper wire as the ion source for ambient ionization mass spectrometry. *Anal. Chem.* 2024, 96, 661–667.
